# Supplementary material for: Retinoids in Fungal Infections: From Bench to Bedside
Source: Pharmaceuticals (Basel). 2021 Sep 24;14(10):962. doi: 10.3390/ph14100962 (PMC8539705; doi:10.3390/ph14100962)
Supplement: Supplementary file 1 [file pharmaceuticals-14-00962-s001.zip › pharmaceuticals-1376482-supplementary.pdf]

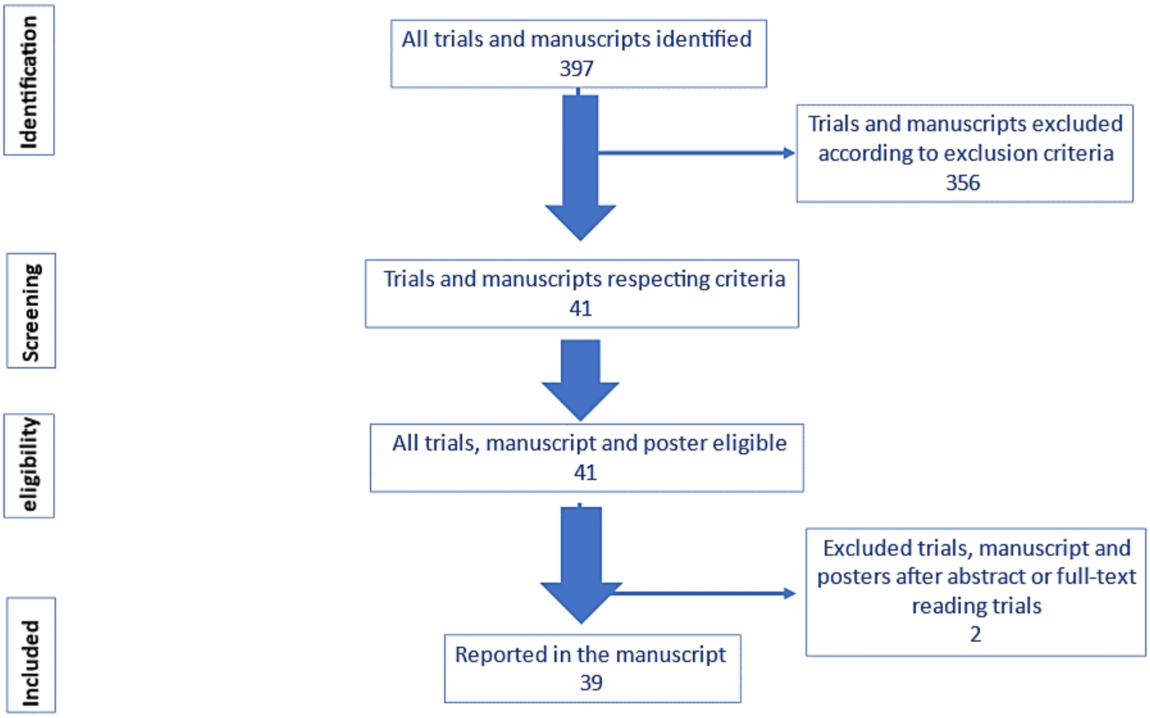

Figure S1. Flow chart reporting the research strategy. Three hundred and ninety-seven article or trials regarding retinoids and fungal infections were identified by this quantitative research. Three hundred and fifty-six were excluded after application of exclusion criteria. Among forty-one article or trials eligible for evaluation, two were excluded after abstract or full text reading. Thirty-nine articles or trials were evaluated in this review.

Table S1. Clinical trial reporting use of retinoid in fungal infection. Accessed on 8th August, 2021. Source: https://clinicaltrials.gov/.

**Official Title on ClinicalTrials.gov or publication title**	**NCT Number, Status**	**Phase**	**Sample size**	**Drugs evaluated**	**Evaluation time**	**Primary Endpoint**	**Results**
Clinical, Laboratorial and Quality of Life Trial to Evaluate the Efficacy and Safety of Low-dose Oral Isotretinoin for Seborrhea	NCT01139749 recruiting	IV	50 participants Randomized Parallel Assignment	oral isotretinoin capsules of 20 mg a day, every other day, for six months	180 days	sebum secretion rate	No result posted
